# Resampling Method for Applying Density-Dependent Habitat Selection Theory to Wildlife Surveys

**DOI:** 10.1371/journal.pone.0128238

**Published:** 2015-06-04

**Authors:** Olivia Tardy, Ariane Massé, Fanie Pelletier, Daniel Fortin

**Affiliations:** 1 Centre d’Étude de la Forêt and Département de biologie, Université Laval, Québec, Québec, Canada; 2 Direction de la biodiversité et des maladies de la faune, Direction générale de l’expertise sur la faune et ses habitats, Ministère des Forêts, de la Faune et des Parcs, Québec, Québec, Canada; 3 Canada Research Chair in Evolutionary Demography and Conservation, Département de biologie, Université de Sherbrooke, Sherbrooke, Québec, Canada; Università degli Studi di Napoli Federico II, ITALY

## Abstract

Isodar theory can be used to evaluate fitness consequences of density-dependent habitat selection by animals. A typical habitat isodar is a regression curve plotting competitor densities in two adjacent habitats when individual fitness is equal. Despite the increasing use of habitat isodars, their application remains largely limited to areas composed of pairs of adjacent habitats that are defined *a priori*. We developed a resampling method that uses data from wildlife surveys to build isodars in heterogeneous landscapes without having to predefine habitat types. The method consists in randomly placing blocks over the survey area and dividing those blocks in two adjacent sub-blocks of the same size. Animal abundance is then estimated within the two sub-blocks. This process is done 100 times. Different functional forms of isodars can be investigated by relating animal abundance and differences in habitat features between sub-blocks. We applied this method to abundance data of raccoons and striped skunks, two of the main hosts of rabies virus in North America. Habitat selection by raccoons and striped skunks depended on both conspecific abundance and the difference in landscape composition and structure between sub-blocks. When conspecific abundance was low, raccoons and striped skunks favored areas with relatively high proportions of forests and anthropogenic features, respectively. Under high conspecific abundance, however, both species preferred areas with rather large corn-forest edge densities and corn field proportions. Based on random sampling techniques, we provide a robust method that is applicable to a broad range of species, including medium- to large-sized mammals with high mobility. The method is sufficiently flexible to incorporate multiple environmental covariates that can reflect key requirements of the focal species. We thus illustrate how isodar theory can be used with wildlife surveys to assess density-dependent habitat selection over large geographic extents.

## Introduction

Density-dependent habitat selection theories hold a central position in evolutionary ecology research, as they can explain spatial distribution patterns of animals and fitness consequences of intra- and interspecific interactions [1, 2]. The ideal free distribution (IFD) and the ideal despotic distribution (IDD) are two such theories. According to IFD, individuals should restrict their distribution to habitats of the highest quality at low densities of conspecifics, but as mean fitness declines with increasing conspecific density in higher-quality habitats, some individuals should be able to obtain equal fitness by moving to lower-quality habitats with relatively few conspecifics [3, 4]. Assuming that individuals are free to choose among habitat patches, individuals living in a mosaic of habitat patches should all end up with the same mean fitness in the landscape through adaptive inter-patch movement decisions. Based on these principles, isodar theory has been designed as a dynamic solution to assess density- and frequency-dependent evolutionary strategies [2].

The fitness consequences of density-dependent habitat selection strategies on spatial distribution of animals can be revealed by using habitat isodars. A habitat isodar is a regression curve that plots individual density in one habitat type against density in a second habitat type at each point where fitness is equal among individuals within and across both habitat types (*e*.*g*., [5, 6]). The intercept of an isodar reveals differences between two habitats in terms of maximal fitness that individuals can attain at a low density of conspecifics [4]. An isodar may have a negative, a positive, or a null intercept. An isodar with an intercept of 0 indicates that there is no selection between the two habitats at a low conspecific density and, therefore, individuals could achieve maximal fitness by being uniformly distributed between habitat types. In contrast, an intercept that is > 0 indicates that maximal fitness in habitat y (*i*.*e*., associated with the Y-axis) exceeds that in habitat x (X-axis) and individuals should more strongly select habitat y than habitat x at a low density of conspecifics. A positive intercept can be due, for example, to higher resource availability in habitat y than in habitat x [4]. The slope represents differences between two habitats in the rate at which fitness decreases as conspecific density increases in each habitat [4].

The slope of a habitat isodar provides insights into density-dependent habitat selection. For example, an isodar with a slope of 1 indicates that individuals do not change their selection between the two habitats as conspecific density increases. According to IFD, fitness should therefore decrease at an equal rate in the two habitats [4]. A slope steeper than 1 indicates that individuals increase their relative use of habitat y as population size increases (see Morris [4] for a representation of habitat isodars with various intercept and slope estimates). According to IFD, fitness should in this case decline more slowly in habitat y with increasing conspecific density. This may reflect disparities in resource quality or habitat structure between habitats that affect resource acquisition, consumption, and conversion into descendants more strongly in habitat x than in habitat y as conspecific density increases [7]. Isodar theory can also be applied in situations where the habitat choice is restricted by territorial behaviors. This principle is the basis of IDD, in which dominant individuals monopolize resources in higher-quality habitats and force subordinate individuals to occupy lower-quality habitats [8]. Subordinates select habitats in a way that equalizes “perceived” fitness in each habitat, but mean fitness differs between habitats [5, 9].

Isodar theory has been successfully applied to numerous taxa, in particular small mammals [10–13], but also birds [14], fishes [15], insects [16], some large-sized mammals [17, 18], and recently, unicellular algae [19]. These isodar studies typically contrast conspecific densities in two adjacent habitats that are defined *a priori*. Many wildlife surveys, such as those employed in most wildlife monitoring programs, are not based on this design. Shenbrot and Krasnov [20] developed a “paraisodar” method that allows density-dependent habitat selection to be assessed along an environmental gradient. In contrast to habitat isodars that use abundance patterns in two habitat types at different times, paraisodars contrast individual densities that are sampled in several habitat types at two different times (*i*.*e*., at low and high densities). However, paraisodars have only been applied over small spatial extents and require several successive surveys.

Here, we propose a resampling procedure that extracts data from wildlife surveys with a design that is suitable for constructing isodars in heterogeneous landscapes without having to predefine habitat types. We then apply this method to evaluating density-dependent habitat selection by two hosts of the raccoon rabies virus variant: raccoon (*Procyon lotor* L.) and striped skunk (*Mephitis mephitis* L.). Raccoon populations act as a major wildlife reservoir of rabies virus in North America, whereas the number of rabid striped skunks tends to increase as the number of rabies cases in raccoons increases [21]. Identification and characterization of areas at high densities of raccoons and striped skunks should increase the efficiency of prevention and control programs [22, 23].

## Materials and Methods

### Random resampling across the survey area

To apply isodar theory to observations from wildlife surveys, independent sampling units need to be randomly placed over the survey area. This process involves two steps: 1) randomly placing blocks over the survey area, and 2) dividing those blocks into two adjacent sub-blocks of the same size where animal abundance will be estimated based on the survey data. A broad range of field methods can be used to estimate animal abundance (*e*.*g*., capture, sightings, and census) [24]. Given these two steps, however, our resampling method would be particularly effective when wildlife surveys are intensive and the allocation of sampling effort is uniform over the study area. With intensive surveys, mark-recapture techniques (as, *e*.*g*., in the oral rabies vaccination programs in the U.S. and North America [25–27] and in Merkle and Fortin [28]) could be used to estimate animal abundance in each sub-block, thereby providing particularly reliable estimates of local abundance. With a uniform allocation of sampling effort, the random blocks are more likely to consistently contain the minimum sampling effort needed to obtain reliable estimates of local animal abundances (the creation of valid sub-blocks [see Step 2 below] would then be relatively simple because each area of the survey region would be sampled with the same intensity). Our approach, however, can be used in more complex settings. Here, we considered a case study where sampling effort varied over the survey area, with live traps being strategically distributed to maximize the number of captures (near streams and roads, at the base of trees) [22] (see section “Survey areas and captures of raccoons and striped skunks” for further details about the capture procedure). The case study thus demonstrates how our method can be applied to non-uniform sampling schemes. We now describe Steps 1 and 2 in detail.

#### Step 1: creating random blocks

**Fig 1:**
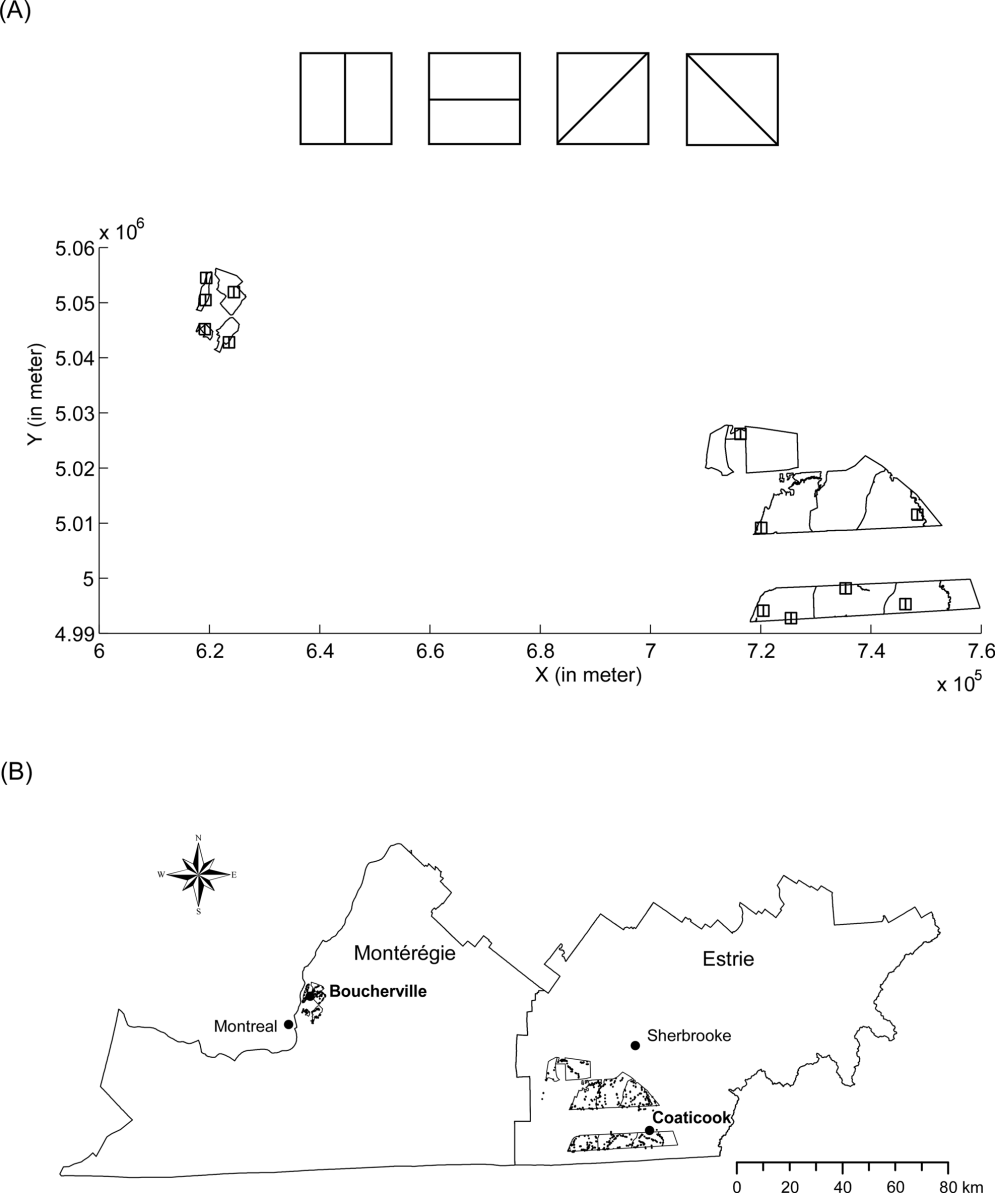
Maps of the survey areas with blocks and associated sub-blocks. (A) Representation of four designs of sub-blocks, *i*.*e*., with horizontal, vertical, and diagonal (right and left) dividing line of sub-blocks. We illustrate below an example of vertical pairs of sub-blocks randomly placed over the survey areas. (B) Map of two survey areas situated in the Montérégie and Estrie regions, Québec, Canada. Trapping sites and live traps (black points) are represented.

The centroids of square blocks are randomly placed across the survey area (Fig 1A). The X and Y coordinates of each centroid are randomly generated from a list containing all possible coordinates that encompass the survey area. As suggested by Tyler et al. [29], the spatial extent over which IFD is applied should correspond to maximum daily movements of animals. Block size can thus be defined from mean home range size of the focal species. The distance between blocks must be determined in such a way that no individual is captured in more than one block. Each block (*i*.*e*., a pair of sub-blocks, see below) should thus be sufficiently spaced apart at a distance that would ensure their independence. This distance should exceed the typical dispersal distance of the species. Further, all blocks should have the same size to avoid a potential correlation between block size and animal abundance.

#### Step 2: creating pairs of adjacent sub-blocks

Each block is divided into two adjacent sub-blocks of the same area in four different configurations (Fig 1A). This approach should reduce the potential risk of biases that are due, for example, to anisotropic patterns in habitat features across the survey area and, hence, in animal abundance.

**Fig 2:**
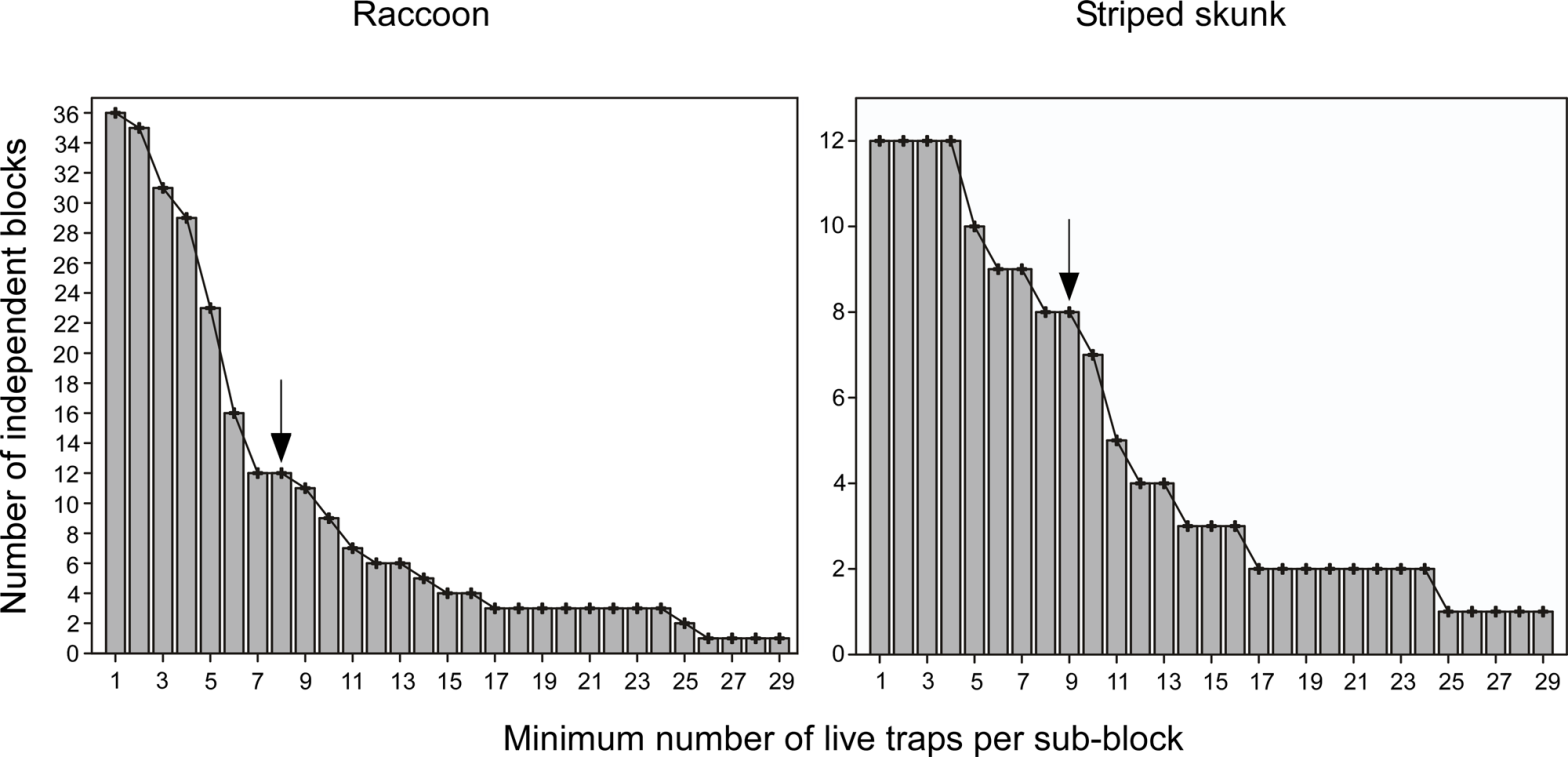
Number of spatially independent blocks placed over the survey areas. Frequency histogram for raccoons (left-hand side) and striped skunks (right-hand side) summarizing the number of independent blocks that can be placed in the survey areas for a predefined minimum number of live traps contained in each paired sub-block in the Montérégie and Estrie regions, Québec, Canada. The point under the arrow represents the favored trade-off that was used to determine the number of blocks to place across the survey areas.

The number of independent blocks that should be placed across the survey area can be determined by visually examining a plot of the number of spatially independent blocks that can be randomly distributed over the survey area against the minimum number of sampling units (*e*.*g*., live traps, sampling plots) that are contained in each paired sub-block (Fig 2). To determine the number of blocks that should be used in the analysis, we randomly place blocks across the survey area according to the protocol defined in the section “Step 1: creating random blocks” and count the number of blocks that satisfies a predefined minimum number of sampling units in each paired sub-block. While the number of blocks has to be sufficiently large to provide a reasonable sample size for regression analysis, the number of sampling units also has to be large enough to estimate the relative animal abundance adequately within the sub-blocks of each pair. An adequate trade-off between these two variables could be found by the inflection point in the relationship between the number of spatially independent blocks as a function of the minimum number of sampling units that are contained in each sub-block. In this way, the entire survey area should be sampled with sufficient intensity to build isodar models.

### Replication

The random placing of blocks and associated sub-blocks (Steps 1 and 2) is performed 100 times for each design of paired sub-blocks (Fig 1A). This level of replication provides the basis for quantifying the variability in animal abundance between the sub-blocks, and consequently, improves statistical inference relative to methods without replication [30]. At each replication, randomly generated blocks that do not respect conditions relative to the minimum number of sampling units in each paired sub-block, and distance between blocks, are removed and substituted by a new block that is randomly placed in the survey area. Blocks without any animals are also removed because they provide no information on density-dependent habitat selection. In this case, the block is substituted by a new one.

### Isodar theory

Morris and Kingston [31] and Hodson et al. [10] demonstrated how to incorporate continuous habitat covariables into density-dependent fitness equations, and subsequently, into isodars. As proposed by Hodson et al. [10], we calculate differences in habitat features (*e*.*g*., measures of habitat disturbance intensity, differences in resource availability and in habitat structure) between pairs of adjacent sub-blocks, and we integrate the related covariates into isodars. If we consider two adjacent sub-blocks named H and L that vary in terms of their habitat features, the isodar then takes this general structure:

$${\text{N}}_{{\text{H}}_{\text{Cov }i}}=\beta_{\text{0}}+\beta_{{\text{N}}_{{\text{L}}_{\text{Cov }i}}}\times {\text{N}}_{{\text{L}}_{\text{Cov }i}}+\beta_{\Delta \text{Cov }i}\times \Delta \text{Cov }i+\beta_{{\text{N}}_{{\text{L}}_{\text{Cov }i}}\times \Delta \text{Cov }i}\times {\text{N}}_{{\text{L}}_{\text{Cov }i}}\times \Delta \text{Cov }i$$ (1)

where sub-block H is the pair member with the highest value for the focal habitat feature *i* (Cov *i*), so that the difference in habitat features between sub-blocks H and L (ΔCov *i*) is necessarily ≥0. ${\text{N}}_{{\text{H}}_{\text{Cov }i}}$ then corresponds to conspecific abundance in sub-blocks H for which the value of the focal habitat feature *i* (Cov *i*) is the highest and ${\text{N}}_{{\text{L}}_{\text{Cov }i}}$ is conspecific abundance in sub-blocks L for which the value of the focal habitat feature *i* (Cov *i*) is the lowest. From Eq 1, several functional forms of density-dependence in habitat selection can be tested because they would be represented by different isodars that vary according to their intercept (equal to β_0_ + β_ΔCov *i*_ × Δ_Cov *i*_) and slope (equal to $\beta_{{\text{N}}_{{\text{L}}_{\text{Cov }i}}}+\beta_{{\text{N}}_{{\text{L}}_{\text{Cov }i}}\times \Delta \text{Cov }i}\times \Delta \text{Cov }i$). In particular, the signs of β_0_ and $\beta_{{\text{N}}_{{\text{L}}_{\text{Cov }i}}}$ inform on the selection for a sub-block type (*i*.*e*., H or L) at low and high conspecific densities in the landscape, respectively. However, the signs of β_ΔCov *i*_ and $\beta_{{\text{N}}_{{\text{L}}_{\text{Cov }i}}\times \Delta \text{Cov }i}$ indicate how the selection for a given sub-block type varies with increasing differences in habitat features between the two types of sub-blocks (ΔCov *i*) at low and high conspecific densities, respectively. For example, if β_0_≥0, $\beta_{{\text{N}}_{{\text{L}}_{\text{Cov }i}}}=1$, β_ΔCov *i*_>0, and $\beta_{{\text{N}}_{{\text{L}}_{\text{Cov }i}}\times \Delta \text{Cov }i}=0$ in Eq 1, individuals should more strongly select sub-blocks H than sub-blocks L at a low density of conspecifics within the landscape, and the selection for sub-blocks H should increase with increasing differences in habitat features between the two types of sub-blocks. If β_0_ = 0, $\beta_{{\text{N}}_{{\text{L}}_{\text{Cov }i}}}>0$, β_ΔCov *I*_ = 0, and $\beta_{{\text{N}}_{{\text{L}}_{\text{Cov }i}}\times \Delta \text{Cov }i}>0$ in Eq 1, individuals should more strongly select sub-blocks H than sub-blocks L at a high density of conspecifics within the landscape, and the selection for sub-blocks H should increase with increasing differences in habitat features between the two types of sub-blocks. If β_0_ ≥ 0, $\beta_{{\text{N}}_{{\text{L}}_{\text{Cov }i}}}>0$, β_ΔCov *i*_>0, and $\beta_{{\text{N}}_{{\text{L}}_{\text{Cov }i}}\times \Delta \text{Cov }i}>0$ in Eq 1, individuals should more strongly select sub-blocks H than sub-blocks L at both high and low densities of conspecifics within the landscape, and the selection for sub-blocks H should increase with increasing differences in habitat features between the two types of sub-blocks. Other forms of isodars are also shown in Hodson et al. [10].

With our method, isodars can be constructed using linear mixed-effects models (LMMs) that consider hierarchical structure in the data (factor “replication” is nested within factor “design of paired sub-blocks”). In Eq 1, ${\text{N}}_{{\text{H}}_{\text{Cov }i}}$ corresponds to conspecific abundance in the sub-block with the highest value for a habitat feature *i* (Cov *i*), and ${\text{N}}_{{\text{H}}_{\text{Cov }i}}$ is systematically assigned to the Y-axis in isodars. Therefore, animal abundance in a given sub-block may be associated with the dependent variable ${\text{(N}}_{{\text{H}}_{\text{Cov }i}})$ of the isodar for a particular habitat feature and with the independent variable ${\text{(N}}_{{\text{L}}_{\text{Cov }i}})$ for another feature. This possibility has some implications for the statistical comparison of candidate isodars. Indeed, an information-theoretic approach [32] can only be used to compare different functional forms of isodars involving a given habitat covariate (ΔCov *i*) because, in this case, the response variable ${\text{(N}}_{{\text{H}}_{\text{Cov }i}})$ does not differ among nested models. However, information theory cannot be used to contrast models involving different habitat covariates [33]. In this case, we can compare models with the same number of parameters based on goodness-of-fit statistics. Marginal and conditional *R²*, as calculated by Nakagawa and Schielzeth [33], appear particularly suitable because they assess the proportion of variance that is explained by the fixed effects and the entire model (*i*.*e*., both the fixed and random effects), respectively. We illustrate the replication-based random resampling method in the following case study.

### Case study

#### Ethics Statement

All observations and surveys were conducted by personnel of the Ministère des Ressources Naturelles et de la Faune du Québec (MRNF) and its partners in a rabies management program. The study was carried out in accordance with the recommendations outlined in the Guide to the Care and Use of Experimental Animals [34]. Animal capture and handling procedures complied with the Agreement on International Humane Trapping Standards (Government of Canada, 1998) and were approved by the Animal Care Committee of the MRNF. All manipulations were performed by qualified wildlife professionals, and all efforts were made to minimize suffering and stress in the animals.

#### Survey areas and captures of raccoons and striped skunks

The study was conducted in the Montérégie region (45°23’23”N, 73°06’05”W), where 104 cases of the raccoon rabies virus variant were reported between 2006 and 2009 [23], and the Estrie region (45°28’54”N, 71°40’05”W) of Québec, Canada. The two regions are characterized by forest patches, agricultural fields, urban and residential zones, and numerous water bodies.

From 27 September to 20 October 2008, raccoons and striped skunks were captured in four trapping sites in the Montérégie region and in eight trapping sites in the Estrie region (Fig 1B) over ten consecutive nights. The live traps (Tomahawk Live Trap, Tomahawk, Wisconsin, USA; Havahart, Woodstream, Lititz, Pennsylvania, USA) were baited with sardines, marshmallows, and an olfactory lure (ProCoon, Leurres Forget, Mauricie, Québec, Canada). A total of 1164 live traps were placed across the two survey areas (334 traps in the Montérégie region and 830 in the Estrie region). Each live trap was inspected daily. Captured animals were immobilized with an injection of ketamine and medetomidine that was administered intramuscularly [35]. Animals were then sexed and marked with ear tags before being released. A handheld Global Positioning System (GPS) location was noted at each installation and removal of a live trap, and at each capture site, to determine trapping effort and number of captures per trap according to its location. Trapping effort was defined as the total number of trap nights deployed minus the number of trap nights where individuals were recaptured and, non-target species were captured. A total of 610 raccoons and 121 striped skunks were captured in October 2008 across the two survey areas.

#### Resampling procedure

We drew blocks of 2 km × 2 km that were spaced at least 2 km apart, given that mean home range size is 2.58 km² for raccoons and 2.32 km² for striped skunks (based on GPS location data, data not shown). No raccoon and striped skunk was recaptured > 2 km from the position of its first capture. From the frequency histogram showing the number of spatially independent blocks that can be placed in the survey areas for a predefined minimum number of live traps contained in each paired sub-block (Fig 2), we noted that when we placed a single block over the two survey areas, it generally fell in an area where both of its sub-blocks had as many of 29 traps. Conversely, when we randomly placed 36 blocks (while respecting the 2-km spacing), some of the sub-blocks had no more than one trap, which is insufficient to obtain a valid estimate of relative animal abundance. For raccoons, we found that by placing 12 blocks, we were able to maintain a minimum of eight live traps per sub-block. For striped skunks, we identified that each of two sub-blocks could encompass at least nine live traps by randomly placing eight blocks over the survey areas (Fig 2). Consequently, we chose to randomly place 12 blocks for raccoons and eight blocks for striped skunks containing at least eight live traps for raccoons and nine live traps for striped skunks across the two survey areas. We calculated relative abundance of raccoons and striped skunks in each paired sub-block as the total number of unique individuals per species captured per 100 trap nights, corrected for trapping effort. In total, 4800 blocks (*i*.*e*., 9600 sub-blocks = 2 sub-blocks/block × 12 blocks × 100 replications × 4 designs of sub-blocks) for raccoons and 3200 blocks (*i*.*e*., 6400 sub-blocks = 2 sub-blocks/block × 8 blocks × 100 replications × 4 designs of sub-blocks) for striped skunks were randomly generated and placed across the Montérégie and Estrie regions using MATLAB software [36]. The relative abundance of raccoons in the 9600 sub-blocks varied from 1 to 52.4 raccoons per 100 trap nights (mean = 14.1), whereas the relative abundance of striped skunks in the 6400 sub-blocks varied from 0.4 to 45.2 striped skunks per 100 trap nights (mean = 8.1).

#### Measures of landscape composition and structure

Landscape composition and structure were measured in each pair of sub-blocks according to land cover types that were delineated on 1:15,000 scale aerial photographs that had been taken in 1999 by the MRNF and information that was obtained from the Financière Agricole du Québec about the different crops that had been cultivated in the agricultural fields. We calculated the proportion of forests, corn fields, wetlands and anthropogenic areas, and quantified the density (km/km²) of forest edges bordering corn fields in each paired sub-block (S1 Table) by using ArcGIS (version 10.0; Environmental Systems Research Institute, Redlands, California, USA) and Geospatial Modelling Environment (version 0.7.1.0; [37]). We retained these environmental covariables because they influence the spatial distribution and abundance of raccoons and striped skunks [22, 38, 39]. Given that habitat covariates were spatially auto-correlated in the landscape, we used principal component analysis (PCA) to reduce the dimensionality of the multivariate data to independent components that characterize environmental gradients. We determined the number of significant PCA axes by using the broken-stick method [40]. The significant PCA axes are those for which the percentage of explained variance is larger than the percentage calculated by the broken-stick distribution [40]. For raccoons, the first principal component (PC1) and the second principal component (PC2) were significant, whereas only PC1 was significant for striped skunks. Accordingly, we used significant PCA axes to characterize each sub-block, for each species.

#### Statistical analysis

To construct isodars, we need to identify the sub-block of each pair with the highest PC1 or PC2 score. We refer to sub-block H of a pair as the one with the highest PC1 or PC2 score (*i*.*e*., sub-block H can be different when identified with PC1 versus PC2 score). Following Hodson et al. [10], we introduced landscape features into the isodars by estimating the difference in landscape composition and structure between sub-blocks H and L along an environmental gradient that was represented by PC1 or PC2 scores: ΔPCi= (PCi score of sub-block H)−(PCi score of sub-block L).

By definition, PC*i* (i = 1 or 2) scores for sub-blocks H were larger than or equal to scores for sub-blocks L and ΔPC*i* ≥ 0. The isodars had the following general structure:

$${\text{N}}_{{\text{H}}_{\text{PC}i}}=\beta_{\text{0}}+\beta_{{\text{N}}_{{\text{L}}_{\text{PC}i}}}\times {\text{N}}_{{\text{L}}_{\text{PC}i}}+\beta_{\Delta \text{PC}i}\times \Delta \text{PC}i+\beta_{{\text{N}}_{{\text{L}}_{\text{PC}i}}\times \Delta \text{PC}i}\times {\text{N}}_{{\text{L}}_{\text{PC}i}}\times \Delta \text{PC}i+\gamma_{i}\times {\text{Z}}_{i}+\gamma_{ij}\times {\text{Z}}_{ij}$$

Where ${\text{N}}_{{\text{H}}_{\text{PC}i}}$ corresponds to conspecific abundance in sub-blocks H that have the highest PCA scores (PC*i*), and ${\text{N}}_{{\text{L}}_{\text{PC}i}}$ is conspecific abundance in sub-blocks L that have the lowest PCA scores. Z_*i*_ represents the random effect for a disposition *i* of sub-blocks and Z_*ij*_ corresponds to the random effect for a replication *j* within a disposition *i* of sub-blocks. The γ are the coefficients associated with each random effect.

For each species and its significant principal component (PC1 or PC2), we tested four candidate isodar models according to Eq 1 (S2 Table). We used linear mixed-effects models, assuming a Gaussian distribution for the response variable. Because the data structure was hierarchical (factor “replication” is nested within factor “design of paired sub-blocks”), the mixed models included a random intercept for each design of sub-blocks (four different designs) to accommodate non-independence of blocks between the different designs and within each design of paired sub-blocks. Also, we considered an exchangeable (or compound symmetry) correlation structure that takes into account the correlation between observations of the same replication within a given design of paired sub-blocks [41]. Adding a correlation structure increased model fit (likelihood ratio [LR] = 54.28, *P* < 0.0001 for raccoon PC1 model; LR = 7.92, *P* = 0.005 for raccoon PC2 model; LR = 38.36, *P* < 0.0001 for striped skunk PC1 model). Further, we included a variance structure to stabilize residual heteroscedasticity by allowing for variance increases or decreases with predicted values [41]. Adding a variance structure also increased model fit (LR = 672.58, *P* < 0.0001 for raccoon PC1 model; LR = 364.41, *P* < 0.0001 for raccoon PC2 model; LR = 717.23, *P* < 0.0001 for striped skunk PC1 model).

Normality of random effects and residuals, together with variance homogeneity, were respected for each PCA model. Rather low multicollinearity (variance inflation factor of each covariate < 10) contributed to making reliable statistical inferences [42]. The isodar estimations were computed with the package *nlme* of R statistical software [43]. We compared the four candidate models for each principal component (PC1 or PC2) by using the AIC (Akaike’s Information Criterion) and AIC weights [32]. When several nested models received similar empirical support (ΔAIC < 2), we applied the principle of parsimony by retaining the model with fewer parameters [32]. We then contrasted the goodness-of-fit of the top-ranking models for each principal component (PC1 or PC2) by calculating the marginal and conditional *R²* [33].

We used a randomization method [44] to insure that the observed relationships from best isodar models for raccoons and striped skunks would not be expected by random chance alone; rather, they would instead have resulted from density-dependent habitat selection. To develop a null model for raccoons, we randomized the 12 pairs of adjacent sub-blocks so that “non-adjacent” sub-blocks were associated in the regression analyses. This process was performed for the 400 times that 12 pairs of adjacent sub-blocks were drawn over the survey areas in the original isodar analyses (*i*.*e*., for each of the 100 replicates performed for each of the four sub-block dispositions). We then followed the same procedure as described in the section “Isodar theory” by identifying the sub-blocks H and L, and by estimating the relationship between the paired sub-blocks (non-adjacent in this case). This entire process was done 200 times, and the results were used to determine the 95% confidence intervals that would be expected by random chance alone. The same method was applied to striped skunk abundance data, with the only difference that individual isodars were based on eight rather than 12 pairs of sub-blocks.

## Results

For raccoons, the first two PCA axes explained respectively 53% and 31% of the variation in landscape attributes within pairs of adjacent sub-blocks. The PC1 axis represented a gradient from corn fields to forests, whereas PC2 reflected a gradient from anthropogenic areas to forests (Table 1). For striped skunks, PC1 (only significant PCA axis) explained 65% of the variation in landscape attributes and defined a gradient from corn fields to anthropogenic areas (Table 1).

**Table 1:** Factor loadings for the first two axes resulting from principal component analysis (PCA) that was conducted on proportions of land cover types and density (km/km²) of corn-forest edges characterizing pairs of adjacent sub-blocks in the Montérégie and Estrie regions, Québec, Canada.

	Raccoon		Striped skunk
Land cover type	PC1: Corn field to forest gradient	PC2: Anthropogenic area to forest gradient	PC1: Corn field to anthropogenic area gradient
Proportion of forests	0.559	0.739	-0.170
Proportion of corn fields	-0.922	-0.058	-0.668
Proportion of anthropogenic areas	0.252	-0.856	0.760
Proportion of wetlands	-0.071	0.102	0.036
Density of corn-forest edges	-0.738	0.331	-0.798

The isodar models including the effect of the corn field—forest gradient (PC1) and the effect of the anthropogenic area—forest gradient (PC2) explained a high proportion of variation in the spatial distribution of raccoons (marginal *R²* = 0.93 and conditional *R²* = 0.99 for PC1 model, and marginal *R²* = 0.95 and conditional *R²* = 0.96 for PC2 model, in S3 Table). Because the marginal and conditional *R²* remain consistently high for the PC1 model, and because PC1 explained more spatial variation in landscape attributes than PC2, we considered the PC1 model as the top-ranking isodar for raccoons. Striped skunk distribution was related to the corn field—anthropogenic area gradient (marginal *R²* = 0.93 and conditional *R²* = 0.96, in S3 Table). Comparison of candidate isodar models based on AIC revealed that raccoon abundance in sub-blocks H ${\text{(N}}_{{\text{H}}_{\text{PC1}}}\text{)}$ along a corn field—forest gradient (PC1) and striped skunk abundance in sub-blocks H ${\text{(N}}_{{\text{H}}_{\text{PC1}}}\text{)}$ along a corn field—anthropogenic area gradient (PC1) depended upon conspecific abundance in sub-blocks L ${\text{(N}}_{{\text{L}}_{\text{PC1}}}\text{)}$, the difference in landscape composition and structure between sub-blocks H and L (ΔPC1), and the interaction between ${\text{N}}_{{\text{L}}_{\text{PC1}}}$ and ΔPC1 (Model 4 in S3 Table). All other candidate isodar models that were based on PC1 received no empirical support (ω*i* < 0.03), in S3 Table). The randomization procedure demonstrates that the spatial distribution of raccoons and striped skunks between adjacent sub-blocks could not be expected due to random chance alone (S1 and S2 Figs).

**Fig 3:**
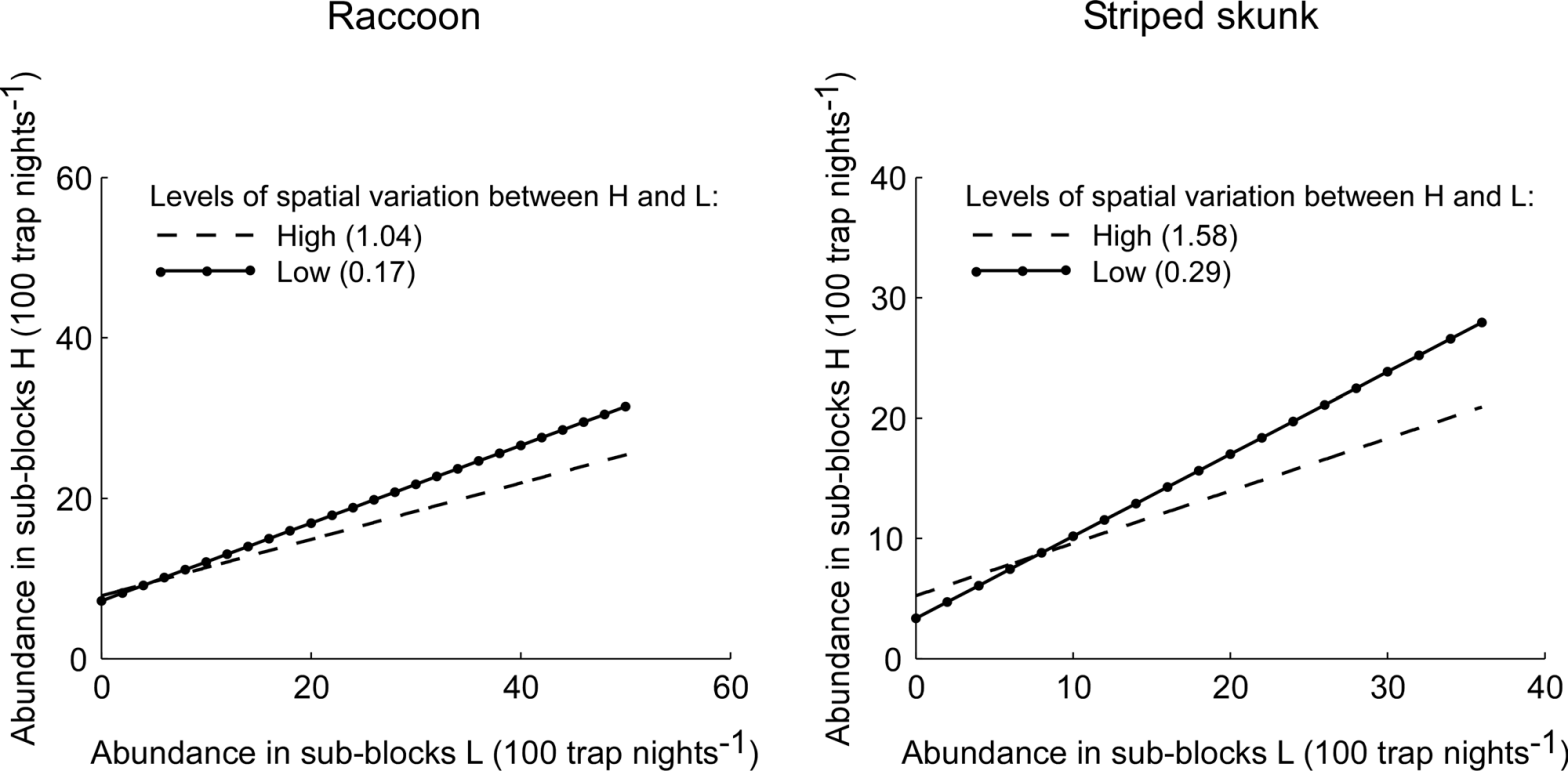
Spatial distribution of species between sub-blocks along an environmental gradient. Estimated isodars for raccoons (left-hand side) and striped skunks (right-hand side) at different levels of variation in landscape composition and structure between sub-blocks H and L along a corn field—forest gradient for raccoons and along a corn field—anthropogenic area gradient for striped skunks. In the raccoon isodar, sub-blocks H correspond to areas with relatively high proportions of forests, whereas sub-blocks L represent areas with rather high densities of corn-forest edges and large proportions of corn fields. In the striped skunk isodar, sub-blocks H define areas with rather high proportions of anthropogenic features, whereas sub-blocks L correspond to areas with relatively high densities of corn-forest edges and large proportions of corn fields. The levels of spatial variation “Low” and “High” represent the values at the 25^th^ and 75^th^ percentiles in the distribution of differences in PC1 scores between sub-blocks H and L (ΔPC1). The percentile values are indicated within parentheses in the figure.

For raccoons and striped skunks, the isodar intercept (equal to β_0_ + β_ΔPC1_ × ΔPC1) was positive within the range of observed values of ΔPC1 (Table 2 and Fig 3), indicating that when conspecific abundance was low in the landscape, raccoons and striped skunks selected areas with relatively high proportions of forests and anthropogenic features, respectively. This selection for these areas increased when the difference in landscape composition and structure between pairs of adjacent sub-blocks was high (β_ΔPC1_ > 0) (in Fig 3).

**Table 2:** Parameter estimates (β) and 95% confidence intervals (CI) for the best isodar model predicting raccoon abundance (a) and striped skunk abundance (b) in sub-blocks H ${\text{(N}}_{{\text{H}}_{\text{PC1}}}\text{)}$ as a function of conspecific abundance in sub-blocks L ${\text{(N}}_{{\text{L}}_{\text{PC1}}}\text{)}$ and the difference in landscape characteristics between sub-blocks H and L (ΔPC1).

Fixed effect	Estimate	95% CI
**a**. Raccoon		
Corn field to forest gradient (PC1)		
$\begin{array}{c} {\text{N}}_{\text{HPC1}}=\beta_{\text{0}}+\beta_{{\text{N}}_{{\text{L}}_{\text{PC1}}}}\times {\text{N}}_{{\text{L}}_{\text{PC1}}}+\beta_{\Delta \text{PC1}}\times \Delta \text{PC1}+\\ \beta_{{\text{N}}_{{\text{L}}_{\text{PC1}}}\times \Delta \text{PC1}}\times {\text{N}}_{{\text{L}}_{\text{PC1}}}\times \Delta \text{PC1} \end{array}$		
β_0_	7.089	[5.841; 8.337]
$\beta_{{\text{N}}_{{\text{L}}_{\text{PC1}}}}$	0.512	[0.475; 0.548]
β_ΔPC1_	0.735	[0.370; 1.100]
$\beta_{{\text{N}}_{{\text{L}}_{\text{PC1}}}\times \Delta \text{PC1}}$	-0.154	[-0.187; -0.122]
**Random effect**		
$\sigma_{\text{inter-disposition}}^{\text{2}}$	1.175	[0.430; 3.208]
$\sigma_{\text{intra-disposition}}^{\text{2}}$	0.502	[0.416; 0.606]
ρ_replication_	-0.034	[-0.041; -0.025]
**b**. Striped skunk		
Corn field to anthropogenic area gradient (PC1)		
$\begin{array}{c} {\text{N}}_{{\text{H}}_{\text{PC1}}}=\beta_{\text{0}}+\beta_{{\text{N}}_{{\text{L}}_{\text{PC1}}}}\times {\text{N}}_{{\text{L}}_{\text{PC1}}}+\beta_{\Delta \text{PC1}}\times \Delta \text{PC1}+\\ \beta_{{\text{N}}_{{\text{L}}_{\text{PC1}}}\times \Delta \text{PC1}}\times {\text{N}}_{{\text{L}}_{\text{PC1}}}\times \Delta \text{PC1} \end{array}$		
β_0_	2.924	[2.087; 3.760]
$\beta_{{\text{N}}_{{\text{L}}_{\text{PC1}}}}$	0.738	[0.684; 0.793]
β_ΔPC1_	1.459	[1.189; 1.729]
$\beta_{{\text{N}}_{{\text{L}}_{\text{PC1}}}\times \Delta \text{PC1}}$	-0.191	[-0.237; -0.144]
**Random effect**		
$\sigma_{\text{inter-disposition}}^{\text{2}}$	0.771	[0.201; 2.952]
$\sigma_{\text{intra-disposition}}^{\text{2}}$	0.849	[0.748; 0.964]
ρ_replication_	-0.046	[-0.059; -0.031]

ΔPC1 is estimated as the difference in PC1 scores between sub-blocks H and L. Sub-block H represents the area with a relatively high proportion of forests (raccoon model) or anthropogenic features (striped skunk model). Sub-block L corresponds to the area with a rather high density of corn-forest edges and a large proportion of corn fields (raccoon and striped skunk models). Variances of random effects between the four designs of sub-blocks ${(\sigma }_{\text{inter-disposition}}^{\text{2}})$ and within each design of sub-blocks ${(\sigma }_{\text{intra-disposition}}^{\text{2}})$, correlation between observations of a same replication within a given design of sub-blocks (ρ_replication_) are shown.

The isodar slope for raccoons and striped skunks (equal to $\beta_{{\text{N}}_{{\text{L}}_{\text{PC1}}}}+\beta_{{\text{N}}_{{\text{L}}_{\text{PC1}}}\times \Delta \text{PC1}}\times \Delta \text{PC1}$) was positive within the range of observed values of ΔPC1 (Table 2 and Fig 3). The slope was less than 1 (Table 2), indicating that at high abundance of conspecifics in the landscape, raccoons and striped skunks favored areas with rather high densities of corn-forest edges and high proportions of corn fields, particularly when the difference in landscape characteristics between pairs of adjacent sub-blocks was high ($\beta_{{\text{N}}_{{\text{L}}_{\text{PC1}}}\times \Delta \text{PC1}}<0$ in Fig 3).

## Discussion

In this study, we developed a resampling procedure that allows isodar theory to be applied over large geographic extents, by contrasting animal densities between pairs of adjacent sub-blocks that are randomly placed within surveyed areas. Using this method, we illustrate how isodar theory can be used with typical wildlife surveys to identify density-dependent habitat selection strategies without having to predefine habitat features. We provide a robust approach for applying isodar theory for a wide range of species, including medium- to large-sized mammals with high mobility. The proposed approach thus has the advantage of being applicable to existing wildlife surveys to reveal the adaptive nature of density-dependent habitat selection for a broad range of wildlife species. In our case study, we used the procedure to identify fitness-rewarding land cover types for raccoons and striped skunks. By revealing behavioral strategies of the main hosts of the raccoon rabies virus variant, the method can delineate areas that are most likely to be occupied by high densities of individuals and, consequently, those that are susceptible to promote rabies virus transmission.

### Resampling procedure to apply isodar theory to wildlife surveys

Generally, studies using classical habitat isodars assess animal densities in multiple sampling plots that are replicated in space along symmetrical line or belt transects over two adjacent habitat types (*e*.*g*., forest versus agricultural field [45]; see Knight and Morris [46] for an illustration of one sampling transect). Accordingly, a pair of adjacent sub-blocks is the experimental unit of the resampling method that we propose to contrast animal densities in areas (sub-blocks) that can be composed of multiple habitat types. In our case, however, the method involves the use of computer modeling to randomly place blocks over the survey area while respecting certain constraints (*e*.*g*., inter-block distance and block size) set by the user. Then, the blocks are divided in two adjacent sub-blocks of the same size, and relative animal abundance is estimated in each sub-block based on the wildlife survey data. Each time blocks (*e*.*g*., 12 blocks for raccoons and eight blocks for striped skunks) are randomly placed over the landscape, we sample only a small portion of the entire survey area because of the constraints that we set, such as maintaining minimum distance between blocks to insure their statistical independence. Nevertheless, because the blocks are replicated several times over the landscape, we end up considering individuals captured over the entire survey area, and we are therefore able to make general inferences about density-dependent habitat selection. Our method is sufficiently flexible to incorporate a broad-range of environmental covariates. Covariates that are considered in developing candidate isodars should still be carefully selected based on the ecology of the focal species, and the resulting isodars should be vetted by ecologists that are familiar with the focal species and the survey area.

The proposed method can be applied over large geographic extents, and provides a helpful framework for wildlife management programs that are based on the broad-scale monitoring of population abundances. Whenever possible, mark-recapture or other types of analysis (*e*.*g*., distance sampling analysis) that incorporate uncertain detection [47, 48] should be used to build isodars. In fact, this point has been recognized in early isodar studies [6]. Most isodar studies, however, do not use mark-recapture data (but see Haugen et al. [49]); instead, they rely upon various estimates of relative abundance. For example, relative animal abundance is often defined as the number of different individuals that are captured (minimum number known to be alive, MNA) during a sampling period [4, 10, 46, 50, 51], or as the fecal pellet deposition rate [10, 17]. In our case, we could not estimate animal abundance from capture-mark-recapture analysis because the number of recaptures was relatively low in each trapping site (raccoons: range = 0–11 and mean = 2.67; striped skunks: range = 0–10 and mean = 2.25), which implies that a large number of sub-blocks contained very few or no recaptures. We thus used the most common approach for building isodars and considered MNA as an index of conspecific abundance.

### Response of raccoons and striped skunks to environmental gradients: implications for rabies virus transmission

In the context of infectious disease dynamics, areas with high host densities must be delineated to increase the efficiency of control and prevention programs [22, 52]. To characterize these areas, most studies examine the relationship between environmental covariates and host abundance patterns (*e*.*g*., [22, 53]), without considering the fitness consequences that are associated with abundance patterns at low and high densities of conspecifics. However, the understanding of adaptive habitat selection can be critical for resolving various problems of wildlife management and conservation [10, 54]. Our method that is based on isodar theory can help identify areas at risk for infectious disease transmission, while permitting the explanation of observed spatial patterns in host densities and their consequences on fitness. To minimize the costs that are associated with vaccination against rabies, densities of oral vaccine baits should be provided in proportion to local host densities [55]. In our study, isodar models suggested that at low abundance of conspecifics in the landscape, raccoons and striped skunks selected areas with rather high proportions of forests and anthropogenic features, respectively. This selection for these areas increased with increasing differences in landscape composition and structure between pairs of adjacent sub-blocks. Both species should achieve maximal potential fitness in areas with high proportions of forests and anthropogenic features, given that these habitats offer larger quantities of resources, such as den sites (*e*.*g*., tree holes for raccoons and burrows under buildings for striped skunks) and occasional food items (*e*.*g*., small mammals, birds, insects, human food, or garbage) [56, 57]. However, in landscapes with relatively high local population sizes, raccoons and striped skunks favored areas with rather high densities of corn-forest edges and high proportions of corn fields, particularly when differences in landscape characteristics between pairs of adjacent sub-blocks were great. Our models predict a lower reduction of potential fitness in these areas when conspecific abundance increases in the landscape. Areas with high densities of corn-forest edges provide raccoons and striped skunks with abundant and highly nutritional food sources from the corn fields and safe cover within the forests [22, 39]. The inter-species transmission risk of rabies virus thus would be highest in the areas with rather high densities of corn-forest edges and high proportions of corn fields, since they support a larger raccoon and striped skunk population. Distribution of oral vaccine baits should be mainly concentrated in these areas, as they are more likely to generate rabies outbreaks due to more frequent contacts among raccoons and striped skunks.

Isodar theory can help to identify ecological traps if fitness components are measured as along with animal densities in two habitats [58]. An ecological trap is an area that animals preferentially select and in which they have lower fitness compared to other available areas [59]. In most cases, ecological traps occur in landscapes that have been degraded by human activities, which alter formerly reliable environmental cues and induce maladaptive habitat choices [60]. To demonstrate the existence of an ecological trap, individuals must show a preference for a trap habitat over other habitats, and must have lower fitness in the habitat trap than in other available habitats [59, 61]. In the case of a disease outbreak, there might be a mismatch between animal density and fitness because disease transmission is often a positive density-dependent process. Pathogenic and parasitic infections can decrease fitness due to their effects on survival and fecundity [62]. In this context, our analysis indicates that areas with high densities of corn-forest edges and high proportions of corn fields could become ecological traps. Indeed, our isodars revealed that when many conspecifics were present in the landscape, raccoons and striped skunks preferred areas with high densities of corn-forest edges and high proportions of corn fields relative to areas with high proportions of forests and anthropogenic features, respectively. Because there is no difference in movement behavior and home range size between rabid and nonrabid individuals [63–65], habitat selection strategies among nonrabid hosts should be representative of those of rabid hosts. Accordingly, mean fitness should be lower for the two species in areas with high densities of corn-forest edges and high proportions of corn fields during an epidemic, because individuals would be more likely to be infected by the rabies virus in these areas due to high densities of conspecifics. By studying density-dependent habitat selection of the main hosts of the raccoon rabies virus variant, our study illustrates how isodar theory can be used to delineate areas of high animal densities where the risk of disease transmission is the greatest.

## Supporting Information

S1 Fig Randomization testing random distribution of raccoons between sub-blocks. Randomization distributions of parameter estimates of the best isodar model for raccoons and their observed values (black vertical line). The 95% confidence intervals (CI) are represented by a red vertical line. For randomization distributions, the measure of 95% CIs is based on the values at the 2.5^th^ and 97.5^th^ percentiles in the randomization distribution.
(TIF)

S2 Fig Randomization testing random distribution of striped skunks between sub-blocks. Randomization distributions of parameter estimates of the best isodar model for striped skunks and their observed values (black vertical line). The 95% confidence intervals (CI) are represented by a red vertical line. For randomization distributions, the measure of 95% CIs is based on the values at the 2.5^th^ and 97.5^th^ percentiles in the randomization distribution.
(TIF)

S1 Table Measures of landscape composition and structure in sub-blocks. Range of land cover type proportions and density of corn-forest edges (km/km²) characterizing pairs of adjacent sub-blocks for raccoons and striped skunks in the Montérégie and Estrie regions, Québec, Canada.
(DOCX)

S2 TableCandidate isodar models.Hypotheses and associated isodar models for raccoons and striped skunks predicting conspecific abundance in sub-blocks H ${\text{(N}}_{{\text{H}}_{\text{PC}i}}\text{)}$ as a function of conspecific abundance in sub-blocks L ${\text{(N}}_{{\text{L}}_{\text{PC}i}}\text{)}$ and the difference in landscape composition and structure between sub-blocks H and L (ΔPC*i*). ΔPC*i* is measured as the difference in scores obtained from principal component analysis (PCA) between sub-blocks H and L in the Montérégie and Estrie regions, Québec, Canada.(DOCX)

S3 TableRelative empirical support for candidate isodars.List of isodar models predicting raccoon abundance (a) and striped skunk abundance (b) in sub-blocks H ${\text{(N}}_{{\text{H}}_{\text{PC}i}}\text{)}$ as a function of conspecific abundance in sub-blocks L ${\text{(N}}_{{\text{L}}_{\text{PC}i}}\text{)}$ and the difference in landscape characteristics between sub-blocks H and L (ΔPC*i*). ΔPC*i* is estimated as the difference in scores obtained from principal component analysis (PCA) between sub-blocks H and L. Sub-block H defines either the area with a relatively high proportion of forests (raccoon PC1 and PC2 models) or the area with a rather large proportion of anthropogenic features (striped skunk PC1 model). Sub-block L corresponds to either the area with a relatively high density of corn-forest edges and a large proportion of corn fields (raccoon PC1 model and striped skunk PC1 model) or the area with a rather high proportion of anthropogenic features (raccoon PC2 model). Number of parameters (K), Akaike’s Information Criterion (AIC), delta-AIC values (ΔAIC), and AIC weights (ω_*i*_) are presented. The selected models are identified in bold with their values of marginal and conditional *R* ^*2*^ (${\text{R}}_{\text{M}}^{\text{2}}$: marginal *R* ^*2*^ and ${\text{R}}_{\text{C}}^{\text{2}}$: conditional *R* ^*2*^).(DOCX)
