# Supplementary figures and images for: Resampling Method for Applying Density-Dependent Habitat Selection Theory to Wildlife Surveys
Source: PLoS One. 2015 Jun 4;10(6):e0128238. doi: 10.1371/journal.pone.0128238 (PMC4456250; doi:10.1371/journal.pone.0128238)

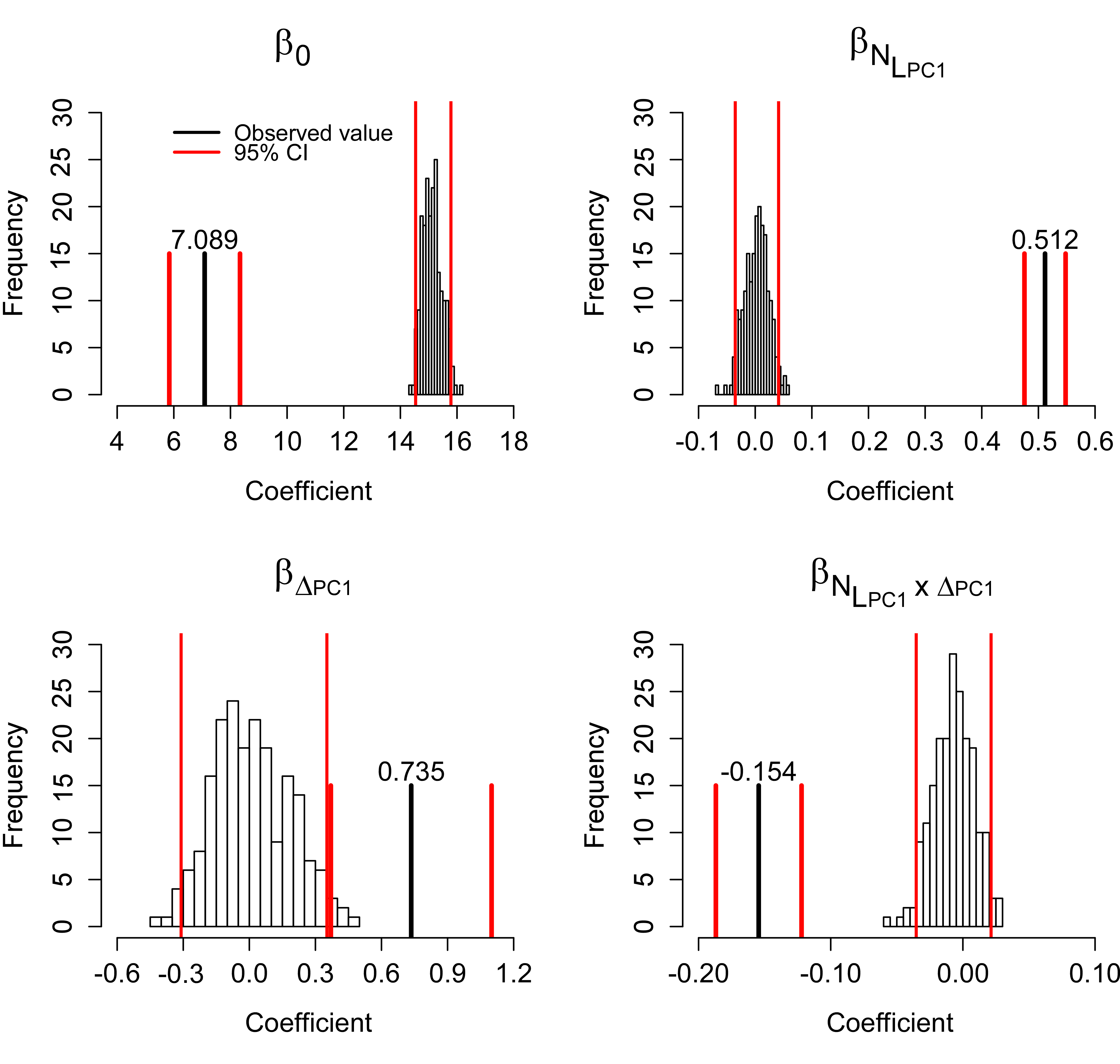

Supplement: S1 Fig — Randomization distributions of parameter estimates of the best isodar model for raccoons and their observed values (black vertical line). The 95% confidence intervals (CI) are represented by a red vertical line. For randomization distributions, the measure of 95% CIs is based on the values at the 2.5th and 97.5th percentiles in the randomization distribution. (TIF) [file pone.0128238.s001.tif]

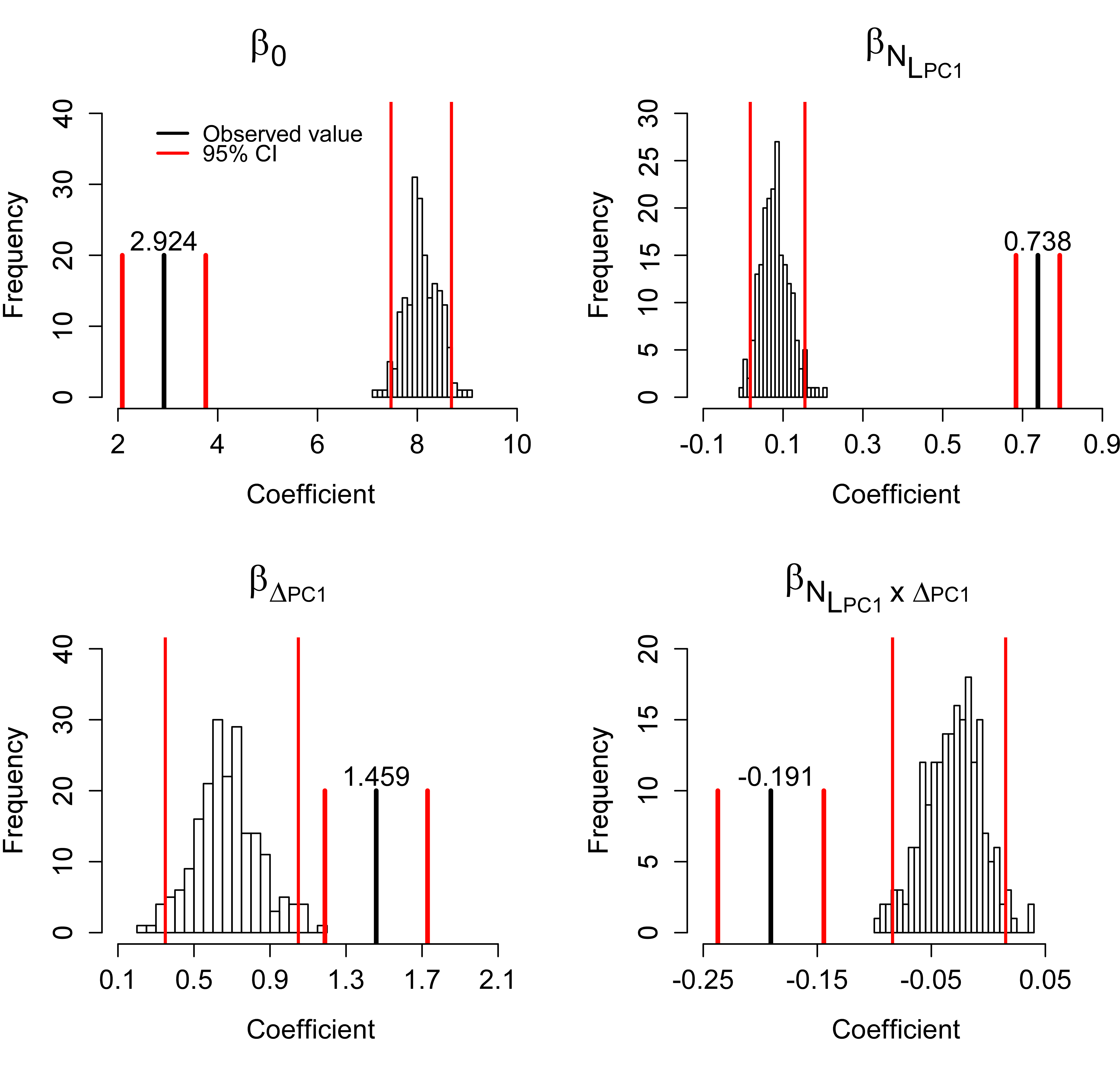

Supplement: S2 Fig — Randomization distributions of parameter estimates of the best isodar model for striped skunks and their observed values (black vertical line). The 95% confidence intervals (CI) are represented by a red vertical line. For randomization distributions, the measure of 95% CIs is based on the values at the 2.5th and 97.5th percentiles in the randomization distribution. (TIF) [file pone.0128238.s002.tif]
