# Supplementary material for: Resampling Method for Applying Density-Dependent Habitat Selection Theory to Wildlife Surveys
Source: PLoS One. 2015 Jun 4;10(6):e0128238. doi: 10.1371/journal.pone.0128238 (PMC4456250; doi:10.1371/journal.pone.0128238)
Supplement: S1 Table — Range of land cover type proportions and density of corn-forest edges (km/km²) characterizing pairs of adjacent sub-blocks for raccoons and striped skunks in the Montérégie and Estrie regions, Québec, Canada. (DOCX) [file pone.0128238.s003.docx]

**S1 Table.** **Measures of landscape composition and structure in sub-blocks.**

Range of land cover type proportions and density of corn-forest edges (km/km²) characterizing pairs of adjacent sub-blocks for raccoons and striped skunks in the Montérégie and Estrie regions, Québec, Canada

|  | **Raccoon** | **Striped skunk** |
| --- | --- | --- |
| Proportion of forests | 0-1 | 0-0.77 |
| Proportion of corn fields | 0-0.61 | 0-0.62 |
| Proportion of anthropogenic areas | 0-0.99 | 0-0.87 |
| Proportion of wetlands | 0-0.46 | 0-0.48 |
| Density of corn-forest edges | 0-2.3 | 0-1.62 |
