# Supplementary material for: Resampling Method for Applying Density-Dependent Habitat Selection Theory to Wildlife Surveys
Source: PLoS One. 2015 Jun 4;10(6):e0128238. doi: 10.1371/journal.pone.0128238 (PMC4456250; doi:10.1371/journal.pone.0128238)
Supplement: S2 Table — Hypotheses and associated isodar models for raccoons and striped skunks predicting conspecific abundance in sub-blocks H (NHPCi) as a function of conspecific abundance in sub-blocks L (NLPCi) and the difference in landscape composition and structure between sub-blocks H and L (ΔPCi). ΔPCi is measured as the difference in scores obtained from principal component analysis (PCA) between sub-blocks H and L in the Montérégie and Estrie regions, Québec, Canada. (DOCX) [file pone.0128238.s004.docx]

**S2 Table. Candidate isodar models.** Hypotheses and associated isodar models for raccoons and striped skunks predicting conspecific abundance in sub-blocks H $\text{(N}_{\text{HPC}i}\text{)}$ as a function of conspecific abundance in sub-blocks L $\text{(N}_{\text{LPC}i}\text{)}$ and the difference in landscape composition and structure between sub-blocks H and L . is measured as the difference in scores obtained from principal component analysis (PCA) between sub-blocks H and L in the Montérégie and Estrie regions, Québec, Canada

| **No.** | **Hypothesis** | **Model** |
| --- | --- | --- |
|  | Effect of environmental gradient (PC*i*) on density-dependent habitat selection |  |
| 1 | No effect |  |
| 2 | Effect at low densities of conspecifics |  |
| 3 | Effect at high densities of conspecifics |  |
| 4 | Effect at low and high densities of conspecifics |  |
