# Supplementary material for: Resampling Method for Applying Density-Dependent Habitat Selection Theory to Wildlife Surveys
Source: PLoS One. 2015 Jun 4;10(6):e0128238. doi: 10.1371/journal.pone.0128238 (PMC4456250; doi:10.1371/journal.pone.0128238)
Supplement: S3 Table — List of isodar models predicting raccoon abundance (a) and striped skunk abundance (b) in sub-blocks H (NHPCi) as a function of conspecific abundance in sub-blocks L (NLPCi) and the difference in landscape characteristics between sub-blocks H and L (ΔPCi). ΔPCi is estimated as the difference in scores obtained from principal component analysis (PCA) between sub-blocks H and L. Sub-block H defines either the area with a relatively high proportion of forests (raccoon PC1 and PC2 models) or the area with a rather large proportion of anthropogenic features (striped skunk PC1 model). Sub-block L corresponds to either the area with a relatively high density of corn-forest edges and a large proportion of corn fields (raccoon PC1 model and striped skunk PC1 model) or the area with a rather high proportion of anthropogenic features (raccoon PC2 model). Number of parameters (K), Akaike’s Information Criterion (AIC), delta-AIC values (ΔAIC), and AIC weights (ωi) are presented. The selected models are identified in bold with their values of marginal and conditional R 2 (RM2: marginal R 2 and RC2: conditional R 2). (DOCX) [file pone.0128238.s005.docx]

**S3 Table**. **Relative empirical support for candidate isodars.** List of isodar models predicting raccoon abundance (a) and striped skunk abundance (b) in sub-blocks H $\text{(N}_{\text{HPC}i}\text{)}$ as a function of conspecific abundance in sub-blocks L $\text{(N}_{\text{LPC}i}\text{)}$ and the difference in landscape characteristics between sub-blocks H and L . is estimated as the difference in scores obtained from principal component analysis (PCA) between sub-blocks H and L. Sub-block H defines either the area with a relatively high proportion of forests (raccoon PC1 and PC2 models) or the area with a rather large proportion of anthropogenic features (striped skunk PC1 model). Sub-block L corresponds to either the area with a relatively high density of corn-forest edges and a large proportion of corn fields (raccoon PC1 model and striped skunk PC1 model) or the area with a rather high proportion of anthropogenic features (raccoon PC2 model). Number of parameters (K), Akaike’s Information Criterion (AIC), delta-AIC values , and AIC weights $\text{(ω}_{i}\text{)}$ are presented. The selected models are identified in bold with their values of marginal and conditional *R²* ($\text{R}_{\text{M}}^{2}$: marginal *R²* and $\text{R}_{\text{C}}^{2}$: conditional *R²*).

| **No.** | **Model** | **K** | **AIC** |  |  |  |  |
| --- | --- | --- | --- | --- | --- | --- | --- |
|  | 1. Raccoon   Effect of corn field – forest gradient (PC1) |  |  |  |  |  |  |
| **4** |  | **8** | **18869.4** | **0.00** | **1** | **0.93** | **0.99** |
| 3 |  | 7 | 18972.2 | 102.86 | 0 | 0.92 | 0.98 |
| 2 |  | 7 | 19076.7 | 207.32 | 0 | 0.91 | 0.98 |
| 1 |  | 6 | 19088.6 | 219.19 | 0 | 0.92 | 0.98 |
|  | Effect of anthropogenic area – forest gradient (PC2) |  |  |  |  |  |  |
| **4** |  | **8** | **31261.7** | **0.00** | **0.972** | **0.95** | **0.96** |
| 2 |  | 7 | 31269.2 | 7.50 | 0.023 | 0.95 | 0.96 |
| 3 |  | 7 | 31272.4 | 10.65 | 0.005 | 0.95 | 0.96 |
| 1 |  | 6 | 31413.6 | 151.92 | 0.000 | 0.92 | 0.93 |
|  | 1. Striped skunk   Effect of corn field – anthropogenic area gradient (PC1) |  |  |  |  |  |  |
| **4** |  | **8** | **18869.4** | **0.00** | **1** | **0.93** | **0.96** |
| 2 |  | 7 | 18972.2 | 102.86 | 0 | 0.92 | 0.95 |
| 3 |  | 7 | 19076.7 | 207.32 | 0 | 0.86 | 0.94 |
| 1 |  | 6 | 19088.6 | 219.19 | 0 | 0.86 | 0.94 |
